# Survey of Ethnoveterinary Medicines Used to Treat Livestock Diseases in Omusati and Kunene Regions of Namibia

**DOI:** 10.3389/fvets.2022.762771

**Published:** 2022-02-22

**Authors:** Ndahambelela Eiki, Matome Maake, Sogolo Lebelo, Bellonah Sakong, Nthabiseng Sebola, Monnye Mabelebele

**Affiliations:** ^1^Department of Agriculture and Animal Health, College of Agriculture and Environmental Sciences, University of South Africa, Pretoria, South Africa; ^2^Department of Life and Consumer Sciences, College of Agriculture and Environmental Sciences, University of South Africa, Pretoria, South Africa

**Keywords:** ethnoveterinary, livestock, health management, diseases, knowledge, Omusati, Kunene, Namibia

## Abstract

The aim of this study was to find, evaluate, and document ethnoveterinary medications used to treat livestock ailments in Namibia's Omusati and Kunene regions. A semi-structured questionnaire was used to interview a total of 100 people. The results of the survey will be useful in creating the groundwork for future scientific research and validation. There were 15 veterinary medicinal plant species identified, which were divided into 10 families. The only types of growth that were utilized were trees, herbs, and bushes. Leaves (71%) were the most widely used plant parts for ethnoveterinary medicine (EVM), followed by bark (14%), stem (8%), and root (7%). Fresh components were frequently preferred in medical compositions. Oral administration was the most common (42.76%), followed by cutaneous (topical) administration (36.18%). Indigenous knowledge was largely passed down through the generations by word of mouth, indicating that it was vulnerable to fragmentation and loss. EVMs were crushed, soaked in water, and administered orally or topically. Farmers who were older had greater EVM knowledge than those who were younger. *Ziziphus mucronate, Combretum collinum*, and *Colophospermum mopane* were used in the treatment of diarrhea. *Z. mucronate* was also used in the treatment of mastitis. Skin infections were treated using *Aloe esculenta* and *Salvadora persica*. *Ximenia americana* and *C. imberbe* were used to treat eye infections in cattle, goats, and sheep. Retained placentas were treated using *Acacia nilotica, A. erioloba*, and *Grewia flavescens*. Roots from *Fockea angustifolia* were used in treating anthrax. *A. esculenta* Leach placed best with a fidelity level (FL) value of 90%, followed by *A. littoralis* Baker in second place (56%), and *Combretum collinum* Fresen in third place (54%). The majority of EVM recipes took 2–3 days to recover. More research is needed to determine the minimum inhibitory concentrations, biological activities, and toxicities, as well as characterize the chemical components of the plants and determine whether there is a plausible mechanism by which plant chemicals or possible physiological effects could achieve the results described by the respondents.

## Introduction

One of the most important income sources in the Omusati and Kunene regions of Namibia is livestock rearing. Livestock as widely known provides local people with calories in the form of meat, milk, and derivatives, as well as a source of income (1). Moreover, it is also a source of employment, manure, and draft power for the cultivation and transport of goods (2) in majority of the developing countries. Farmers in the Omusati and Kunene regions regard cattle farming as a symbol of riches and honor, as well as a precaution against crop failure during droughts (3). These farmers also keep cattle on hand for special occasions like weddings, funerals, and christenings (4). Despite livestock production's contribution to the livelihoods of people in these regions and the world over, its development is inhibited by different constraints (5). Diseases are one of the most significant constraints to cattle productivity (6).

Ethnoveterinary therapies provide the best choices to farmers in Namibia's Omusati and Kunene areas during challenging economic times, when their purchasing power is insufficient to afford ever-increasing veterinary prices. Furthermore, ethnoveterinary medical knowledge is in peril because it is reliant primarily on the collective memories of a few community practitioners (7). The situation is even more dire when it comes to ethnoveterinary medical knowledge, which is limited to a small number of livestock owners. As a result, it is critical to chronicle this ethnoveterinary medical knowledge to pass it along to future generations. Furthermore, identifying therapeutic plants would contribute to the development of ways for protecting and conserving endangered species. It will also help with the creation of herbal gardens, which will help to preserve biodiversity. Ethnoveterinary medical knowledge must be documented to aid in the finding of innovative drug sources (8).

Importantly, forests are necessary for the survival of ethnoveterinary medicine (EVM) therapeutic plant species in the Omusati and Kunene regions. Therefore, documenting the state of indigenous flora would aid in public awareness campaigns about endangered species and sustainable plant-collecting methods. Keeping these facts in view, the study was initiated, to document ethnoveterinary practices used for treating livestock diseases in two selected regions of Namibia. Thus, the study's findings will be utilized to educate communities on the importance of EVM in providing primary livestock healthcare. It will also aid in disseminating the study's livestock healthcare expertise to potentially influence policy change in favor of incorporating favorable EVM practices into national livestock healthcare systems. Further to this, the outcome will also help in raising a local awareness of the importance of medicinal plant conservation and participation in the propagation of threatened species. Agricultural extension officials and veterinary officers will make the entire study available to the public. Hence, the purpose of this study was launched to describe ethnoveterinary approaches for treating livestock infections in the Omusati and Kunene regions of Namibia.

## Materials and Methods

### Study Area

**Figure 1:**
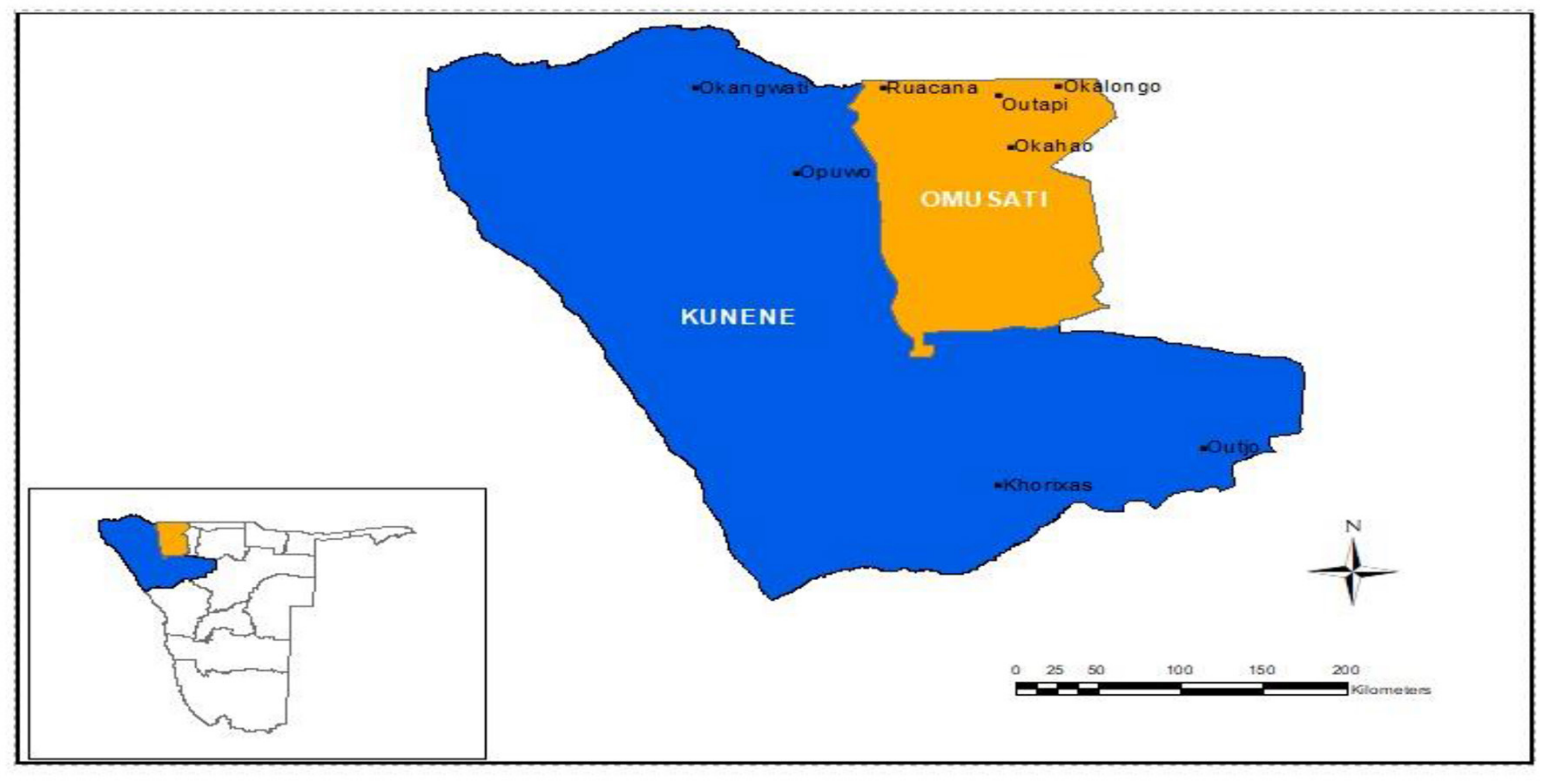
Map of the study area.

The research was carried out in Namibia's Omusati and Kunene areas (see Figure 1). Omusati and Kunene regions border the Kunene River along the Angolan border in northern Namibia. Otjiherero and Nama/Damara languages are spoken by 46 and 36% of people in Kunene, respectively, while Oshiwambo is spoken by 96% of people in Omusati (9). People who speak Otjiherero are generally from the Herero tribe, whereas those who speak Oshiwambo are from the Ovambo tribe. The Bantu tribes found in the study region include the Herero and Ovambo, whereas the Nama/Damara (commonly known as Hottentots or San) are non-Bantu. Aawambo (Ovambo), Ovaherero-speaking pastoralists (Ovahimba, Ovatjimba, and Ovazemba), and Nama/Damara are some of the ethnic groups found in the regions.

The people's culture in the study area is diverse due to the tribal diversity. Men are recognized as the head of the household in Ovambo and Nama/Damara cultures, and they are responsible for cattle and household decision-making (10). In the Ovambo and Nama/Damara tribal groups, men also hunt game animals for meat, build huts for family living, cultivate agricultural land, and provide water for the family. In addition, Damara men oversee agriculture planting and harvesting. Damara and Ovambo women, on the other hand, are responsible for cooking and other homework [Ambunda & de Klerk (10). Pastoralists make up a large portion of the Damara population. The most common religions in the study region are Christianity and African traditional religions.

The Omusati and Kunene regions are largely rural, with crop and animal farming serving as the primary source of income for most residents. The Omusati and Kunene aboriginal peoples rely on animal husbandry as their primary source of income. As a result, the two zones are overgrazed and degraded, with a low diversity of plant species (2). The Omusati and Kunene regions are mostly impoverished, and many villages are isolated due to a lack of proper roads, making it difficult for residents to get modern veterinary services (11). Furthermore, modern veterinary facilities and services are primarily found in cities far from farming villages.

As a result, the Omusati and Kunene peoples have created their own treatment method for most diseases that plague their domestic animals. As a result, these two regions constitute an excellent research model for documenting and distributing EVM knowledge to assist and exchange information to improve basic animal healthcare. The most frequent livestock in the research region is cattle, goats, and sheep.

### Sampling Procedures and Data Collection

Despite EVM's popularity among rural communities in Omusati and Kunene, the number of farmers who use it in either region has not been formally verified. As a result, the researchers had no notion who the study population (N) was before data collection. Snowball sampling was utilized as a result, with key informants included. With the cooperation of extension officials, farmers, and community leaders in both regions, 100 farmers with EVM expertise were identified. Fifty (50) EVM experts were therefore identified in the Omusati region. Despite the difficulty in obtaining the requisite sample size due to geographical limits, the researcher also chose 50 EVM specialists in the Kunene region. Therefore, 50 EVM experts were chosen from each region (Omusati and Kunene).

The Aawambo ethnic group is made up most of the participants, followed by Herero-speaking pastoralists (Ovahimba, Ovatjimba, and Ovazemba), San, and Damara/Nama. Only five women were interviewed among the hundred EVM experts [Omusati (*n* = 45 men and 5 females) and Kunene (*n* = 50 males)], which could be due to men's dominance in livestock raising in the study area. All the EVM experts interviewed were Christians. Three percent (3%) of participants were between the ages of 41 and 50, followed by 69% of those between 51 and 60 and 28% of those over 60 (>60).

Forty-nine percent (49%) of the participants had no formal education, 41% had basic education (primary and secondary education), and 7 and 3% had tertiary education and other educational attainments, respectively. All participants raised cattle, with 86% raising chickens, 83% goats, 4% pigs, and 45% raising other livestock. To collect data for the current study, face-to-face interviews were done utilizing a semi-structured questionnaire.

The varieties of plants used for EVM, as well as the preparation and treatment techniques for EVM, were all gathered. All types of EVM plants were also collected for identification and documentation. Information such as habitat data, a general description of the plant, the collection's geographical location, and the collector's initials were recorded during the data collection procedure. For taxonomic identification, plant specimens were taken from the vegetative component, leaves, floral, fruiting, and/or seed sections, as appropriate. The specimens were labeled with their vernacular names and transported in plastic bags to avoid drying.

### Data Analysis

Codes, themes, and indicators were used to analyze the qualitative data from open-ended questions that required respondents to articulate themselves. The quantitative data collected were entered on a Microsoft Excel database and analyzed using IBM Statistical Package for the Social Sciences (SPSS) version 27. In SPSS, descriptive statistics, mainly frequencies, were used to analyze categorical data. The data for types of plant species collected were analyzed using literature for identification purpose. The voucher specimens were identified after conferring with experts at the Department of Forestry in Windhoek and studying the *Namibian Plants Red Data Book* (12). The fidelity level (FL) was used to measure the importance of ethnoveterinary medicinal species for a certain purpose. The following formula was used to calculate the FL index:


$$\begin{array}{l} FL\left(\%\right)=NP/N\times 100 \end{array}$$

*N* is the total number of uses cited for every given species, and *NP* is the number of cited species for each condition group.

## Results

### Plant Families Used as Ethnoveterinary Medicine

**Figure 2:**
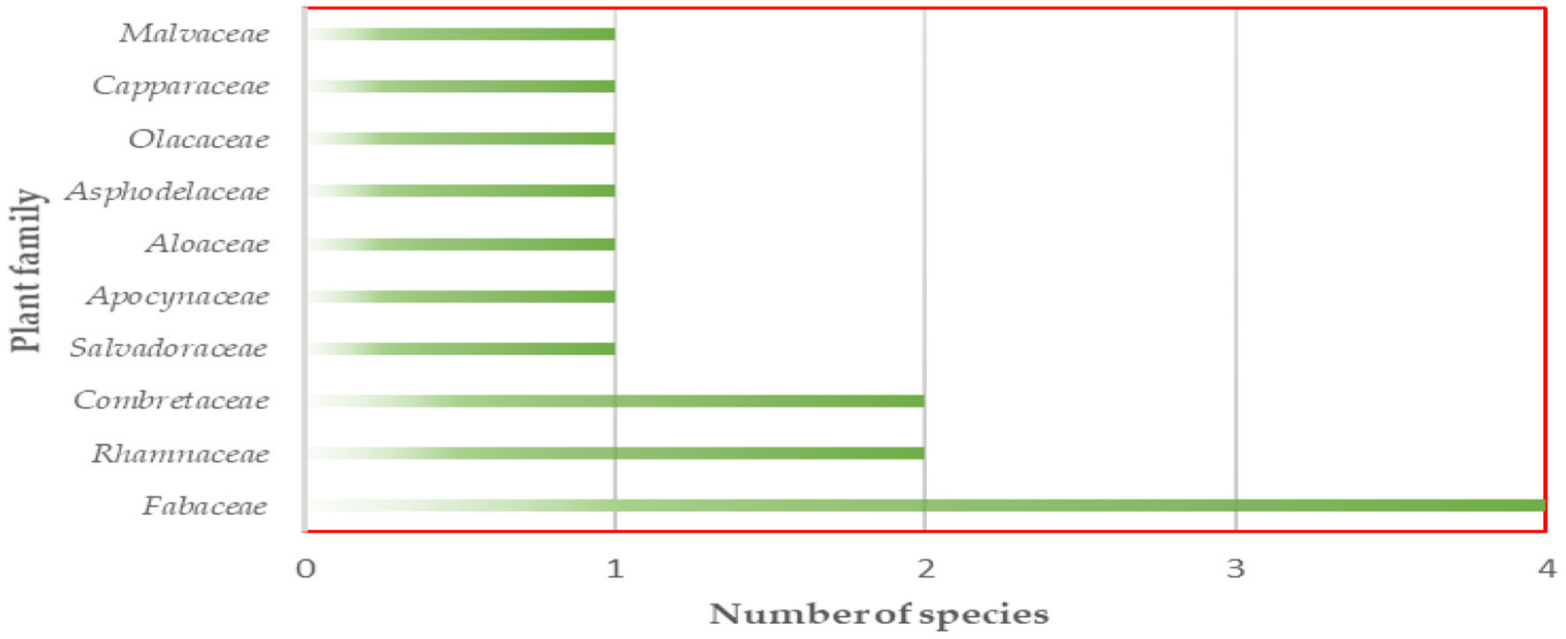
Plant families and number of plant species used in EVM in Omusati and Kunene regions of Namibia.

According to the findings, 15 medicinal herbs were utilized for EVM in the Omusati and Kunene regions (see Table 1). The therapeutic plants identified belonged to 10 different plant families, with Fabaceae being the most common, followed by Combretaceae and Rhamnaceae (see Figure 2). It demonstrates that in the research region, livestock diseases are treated with a variety of plant species.

**Table 1:** Plant species used as ethnoveterinary medicines in Omusati and Kunene regions, Namibia.

**Family**	**Botanical name**	**Common name**	**Voucher number**	^*^Habit	**^#^Habitat**	***^†^*Parts used**	**Preparation used**	**Disease treated**	^∧^ **FL (%)**	**Application method**
Aloaceae	*Aloe esculenta* Leach	Aloe	KHAW2020NE	S	W	L	Fresh leaves infusion	Skin infections and	90.0	Topical
								coughs	28.0	Oral
Apocynaceae	*Fockea angustifolia* K. Schum	Water rootkambroo	KUNW2020NE	H	W	R	Root powder	Anthrax	18.0	Oral
	*Aloe littoralis* Baker	Mopane aloe	KUNW2020NE	T	W	L	Leaf Infusion	Unthriftiness in poultry	56.0	Oral
Combretaceae	*Combretum collinum* Fresen.	Bicolored bushwillow	OMUW2020NE	T	W	S & B	Stem/bark infusion	Constipation, diarrhea, and colic	54.0	Oral
	*Combretum imberbe* Wawra	Lead wood	OMUW2020NE	T	W	L/R/St	Leaf powder decoction	Eye infection	6.0	Topical
								Diarrhea	9.0	Oral
Capparaceae	*Boscia albitrunca* Burch.	Shepherd tree	OMUW2020NE	B/T	W	T/L/R	Decoction	Helminths and lung and liver infections	31.0	Topical
Fabaceae	*Acacia erioloba* E.Mey.	Camel thorn	KUNW2020NE	T	W	B	Bark infusion	Retained placenta	26.0	Oral
	*Acacia nilotica* (L.) Wild	Thorny acacia	OMUW2020NE	T	W	B	Branch infusion	Retained placenta	22.0	Oral
Fabaceae	*Acacia karoo* Hayne.	Sweet thorn	KUNW2020NE	T	W	R	Root infusion	Coughs	34.0	Topical
								Eye inflammation	26.0	Topical
	*Colophospermum mopane* (J.Kirk ex Benth.)	Mopane	OMUW2020NE	T	W	B and L	Bark infusion and decoction of leaves	Diarrhea	14.0	Oral
Malvaceae	*Grewia flavescens* Juss. var. flavescens	Donkey-berry	KUNW2020NE	S/T	W	L and R	decoction	Retained placenta	44.0	Oral
Olacaceae	*Ximenia americana* L.	Tallow wood	OMUW2020NE	T	W	L	Leaf infusion	Eye infections	26.0	Topical
						R and B	Roots/bark powder	Wounds	50.0	Topical
Rhamnaceae	*Berchemia discolor* (Klotzsch) Hemsl.	Brown ivory	OMUT2020NE	T	T	B	Bark infusion	Calf weakness	48.0	Oral
	*Ziziphus mucronata* Willd.	Buffalo thorn tree	OMUW2020NE	T	W	L	Leaf paste	Diarrhea	17.0	Oral
								Mastitis	9.0	Topical
Salvadoraceae	*Salvadora persica* L.	Toothbrush tree	KUNW2020NE	T	W	B and St	Bark/stem infusion	Skin infections	45.0	Topical

* *Plant habitat: S, shrub; T, tree; H, herb;*

† *Part used: R, root; B, bark; L, leaves; St, stem;*

# *Plant habitat: W, wild; T, terrestrial*

∧ *FL, fidelity level*.

**Figure 3:**
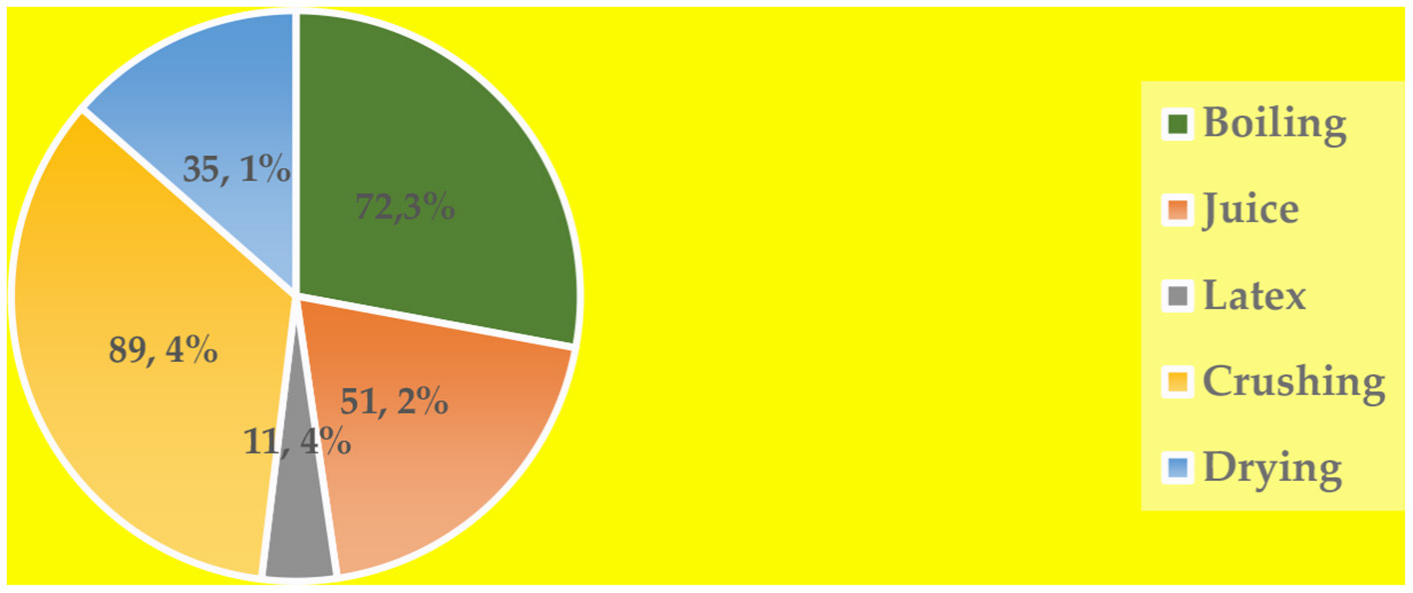
EVM preparation methods used in Kunene and Omusati regions, Namibia (n = 100).

Trees, shrubs, and herbs were among the ethnoveterinary medicinal plant species. Most of the therapeutic plants were found in the wild, with only a few species found on farms (terrestrially). There were no medicinal plant species cultivated. The most part of the plant used for EVM was leaves (71%), followed by bark (14%), stem (8%), and root (7%). With regards to preparation methods for EVM, crushing was mostly utilized (89.3%), followed by boiling (72.3%), juice (51.2%), drying (35.1%), and latex (11.4%) (Figure 3).

**Figure 4:**
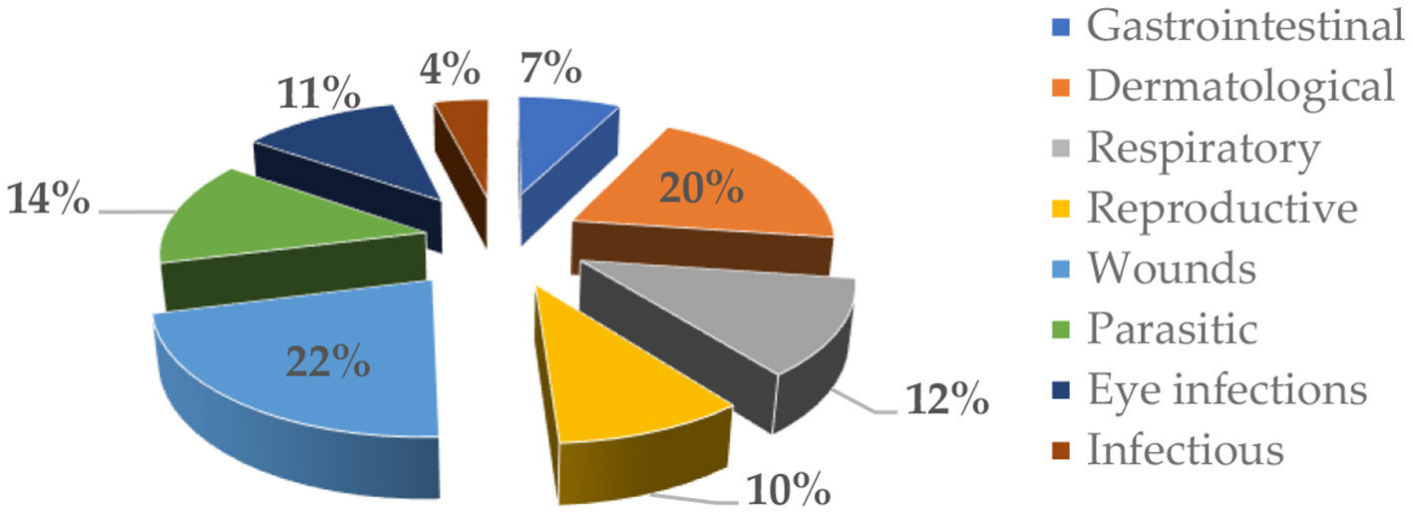
Proportion of veterinary diseases treated with EVM in Omusati and Kunene regions of Namibia (n = 100).

The most prevalent treatment for EVM was for wounds (22%), followed by dermatological issues (20%) and parasitic diseases (14%). Moreover, EVM was also used to treat respiratory diseases (12%), ocular infections (11%), gastrointestinal diseases (7%), and infectious (anthrax and mastitis) diseases (4%) (Figure 4). The majority of the EVM plant materials were utilized fresh, while dried plants were pulverized into a powder and stored in airtight containers. The recovery time for majority of EVM treatments was 2–3 days as shown by 59% of the respondents, followed by 4–5 days (41%).

FL values ranged from 6 to 90%. *Aloe esculenta* leach had the greatest FL value (90%), followed by *A. littoralis* Baker in second place (56%), *Combretum collinum* Fresen in third place (54%), and *Ximenia americana* L. in fourth place (50%) (Table 1). Moreover, *Combretum imberbe* Wawra had a lowest FL value of 6%. Generally, the results showed that most plants had an FL value of <50%.

### Status of Ethnoveterinary Medicinal Plants

Majority of respondents (59%) feel that medicinal plant populations are dwindling, while 35% believe that species are still scarce and 6% believe that nothing has changed (Table 2). The therapeutic plant species are declining, according to the respondents, because they are harder to get by than in previous years.

**Table 2:** status of EVM plant species.

**Items**	**Regional counts (%)**		**Combination (%)**
	**Omusati**	**Kunene**	
Declining	29	30	59
Sparsely available	19	16	35
No change	2	4	6

## Discussion

In the Kunene region, all EVM practitioners were men (100%), whereas in Omusati, 45% of EVM practitioners were men and 5% were women. Similarly, EVM is mostly performed by men in Pakistan's Kohat district, according to (13). This observation was attributed by Taliq et al. (13) to the fact that men are more favored in a shift of knowledge, while women are consigned to family care in most cultures. The fact that males made up the majority of ethnoveterinary practitioners in the study area contradicts (14), who found that female herbal practitioners predominate in Northwest (NW) Yunnan, China.

The outcomes of the study revealed that most respondents had a lower level of education, supporting the idea that less educated people are less acculturated and hence have a greater understanding of conventional remedies (15). This situation is unsustainable since it may be difficult for less educated persons to document the adoption of such methods for future reference. Ethnoveterinary practitioners require some education to acquire specialized skills that may be valuable, such as natural resource conservation and management. It is critical that educated members of the community support ethnoveterinary medicines in the future.

The present study showed that ethnoveterinary medicines were used more by people in the range of 51–60 years old than by the younger generation. These findings corroborate the findings of other researchers who found that knowledge of ethnoveterinary medicine is primarily limited to the elderly in communities (16–19). This narrative where the elderly people are the guardian of ethnoveterinary medicine and at the forefront of its use presents a risk to EVM (20) contended that it is not sustainable to place EVM knowledge in the elderly because it is susceptible to and maybe threatened by death. As a result, immediate documenting of ethnoveterinary medicine practices in Namibia's Omusati and Kunene areas is essential to preserve such knowledge before it is lost forever.

The majority of ethnoveterinary practitioners learned EVM from their parents or grandparents, according to the findings of this study. These findings agreed with those of (21–23). This is unsustainable because undocumented information may be lost when these individuals pass away. The goal of this survey was to gather and preserve EVM knowledge before it becomes obsolete.

The current research identified 15 plant species from 10 families that have veterinary use. However, this number is significantly less than the 46 species reported by (8) in Southern Ethiopia and the 31 plant species reported by (20) in north central Nigeria. Most of the species in EVM came from the Fabaceae family, which contributed four species. The dominance of the Fabaceae family shown in this study is consistent with previous research (23–26).

Ethnoveterinary practitioners make EVM medicines with components from trees, bushes, and herbs. This could be owing to their relative abundance in the research region compared to other habits. The current study's findings, however, contrast with those of (24) who reported shrubs and climbers as suppliers of EVM materials in their study. The respondents gathered medicinal substances from the wild, except for *Berchemia discolor* (Klotzsch) Hemsl., which has a terrestrial habitat. Collecting medicinal materials from the wild is problematic, according to (25), because two-thirds of the world's 50,000 medicinal plants are harvested from the wild, and one-fifth of them are currently threatened. The results of the current study will be critical in raising the knowledge of optimal propagation procedures for these plant species to ensure their long-term survival for sustainable use.

Leaves (71%) were the most used plant materials, followed by bark (14%), stem (8%), and root (7%). This can be explained by the fact that leaves, unlike other parts, such as subterranean organs and plant exudates, take less work to collect when compared to underground parts, stems, bark, and complete plants (26). Many arguments may be made for why leaves are preferred in EVM. They are the part of the plant where photosynthesis takes place, and they contain many physiologically active secondary metabolites that the plant uses to protect itself against herbivores, pests, and diseases (20). Moreover, the fact that leaves were the most used plant parts in this study is a more viable approach than using roots or whole plant, which can endanger the plants' existence (27). However, the findings of this study contrast those of a study conducted by (24) in which roots were reported to be the most often-requested resources. The results also demonstrate that most medicines (89.34%) were manufactured by crushing, a process that is widely used in the preparation of remedies throughout Africa (28–31). It is possible that the ease with which crushing is used in the production of remedies has something to do with it. Oral and topical administration were the only modes of administration described in this study. This corresponds to the results of a study done by (17).

The therapeutic indication of ethnoveterinary remedies in the current study area covered all livestock species. However, EVM were more used for cattle, goat, and chicken diseases. This discrepancy may be due to the richness and how livestock species are valued in the study area rather than the therapeutic range of medicinal plants themselves. Most therapies were used for the treatment of wounds, followed by a gastrointestinal disease characterized by diarrhea. Three plants (*Acacia nilotica, A. erioloba I*, and *Grewia flavescens* Juss.) from the same family were used in the treatment of retained placenta. *Ziziphus mucronate* and *A. karroo* were used in the treatment of diarrhea. *Z. mucronate* was also used in the treatment of mastitis. These findings agree with a study conducted in Ethiopia by (32). EVM practitioners reported using roots from *Fockea angustifolia* in treating cattle suffering from anthrax. However, a study conducted by (18) noted the use of fresh roots from *F. angustifolia* in drawing out poison to snakebites and stings. *X. americana*, and *C. imberbe* were used to relieve eye infections in cattle, goats, and sheep. The use of *X. americana* and *C. imberbe* was also reported by (8, 17, 33). Leaves from *Boscia albitrunca* were used as a cold infusion for treating inflamed eyes of cattle. The recovery time for majority of EVM treatments in the current study was 2–3 days. Similar findings were also reported by (8, 33).

High fidelity (FL) values are highly important in the selection of specific plants for further search of bioactive chemicals, according to (34). The maximum FL is always achieved by widely utilized medicinal plant species. Different plants, such as *A. esculenta* Leach, *A. littoralis* Baker, *C. collinum* Fresen, and *X. americana* L., scored the highest fidelity values and should be subjected to further phytochemical and pharmacological investigation to prove their medicinal efficacy, according to the current study.

EVM practitioners noted that *Salvadora persica* L., *B. albitrunca* Burch., *F. angustifolia* K. Schum, and *G. flavescens* have become scarce in the Omusati and Kunene regions, and some of them are no longer found in their natural habitats. It is unclear whether these four species were simply uncommon or were kept secret by EVM practitioners. More research is needed, particularly ecological investigations in areas of Omusati and Kunene that were inaccessible during this study. To maintain long-term supplies, the conservation status of these species must be reviewed further.

When it came to respondents' knowledge of diminishing medicinal plants, the majority of EVM practitioners were aware that wild medicinal plants are dwindling. Some EVM practitioners, however, stated that medicinal plants are not endangered if it rains. This could indicate that rainfall plays an essential role in the survival of plants rather than exploitation. To ensure their protection, future wider usage, and the preservation of information about their use, it is necessary to properly identify the most endangered and threatened species in Namibia's Omusati and Kunene districts. As a result, the findings of this study can be used to develop threatened species conservation strategies.

## Conclusion

Farmers in the Omusati and Kunene regions, according to this study, have sound ethnoveterinary knowledge and practices. As a result, 15 species having veterinary use have been identified. Plant parts, the methods of preparation, and the sources of such plants were all presented in detail. Because ethnoveterinary medicinal plants are not owned by individual farmers, they may not be properly conserved in the wild, and as a result, they may be lost to deforestation and overexploitation. Therefore, the cultivation of awareness among ethnoveterinary practitioners is one of the most critical approaches for the preservation of these indigenous medicinal plant species. Plants with a high FL should be investigated further for phytochemical analysis and pharmacological activity. In Omusati and Kunene, ethnoveterinary medicinal prescriptions should be examined further to see whether there are any active ingredient interactions and what clinical implications they have.

## Data Availability Statement

The original contributions presented in the study are included in the article/supplementary material, further inquiries can be directed to the corresponding author/s.

## Ethics Statement

The studies involving human participants were reviewed, approved, and conducted in accordance with the University of South Africa's (UNISA) Ethics code for people's participation and plant specimens in research with ethics reference number 2020/CAES_HREC/025.. The patients/participants provided their written informed consent to participate in this study.

## Author Contributions

NE conceived and designed the study, conducted the survey, and finalized the submitted version. MaM, SL, BS, NS, and MoM were involved in data analysis and the drafting of manuscripts. All authors contributed to the article and approved the submitted version.

## Conflict of Interest

The authors declare that the research was conducted in the absence of any commercial or financial relationships that could be construed as a potential conflict of interest.

## Publisher's Note

All claims expressed in this article are solely those of the authors and do not necessarily represent those of their affiliated organizations, or those of the publisher, the editors and the reviewers. Any product that may be evaluated in this article, or claim that may be made by its manufacturer, is not guaranteed or endorsed by the publisher.

## Abbreviations

EVM: ethnoveterinary medicines
S: shrub
T: tree
H: herb
R: root
B: bark
L: leaves
St: stem
W: wild
^∧^FL: fidelity level.
